# Relationship between internalizing and externalizing symptoms trajectories and perinatal risk factors in an epidemiological sample: Preliminary results from the remind project

**DOI:** 10.1192/j.eurpsy.2021.406

**Published:** 2021-08-13

**Authors:** S. Grazioli, E. Rosi, F. Villa, M. Mauri, P. Brambilla, C. Bonivento, M. Molteni, M. Nobile

**Affiliations:** 1 Developmental Psychopathology, Scientific Institute Eugenio Medea, Associazione La Nostra Famiglia, Bosisio Parini, Italy; 2 Department Of Pathophysiology And Transplantation, University of Milan, Milan, Italy; 3 Associazione La Nostra Famiglia, Scientific Institute, IRCCS E. Medea, Pasian di Prato, Italy

**Keywords:** perinatal risk factors, internalizing psychopathology, externalizing psychopathology, psychopathology trajectories

## Abstract

**Introduction:**

Our 15-years follow-up ReMIND project aims to re-assess an epidemiological and a clinical sample of adults (Wave 3), who were assessed in preadolescence (Wave 1) and adolescence (Wave 2), to evaluate symptoms trajectories and their relationship with genetic/epigenetic data, environmental risk factors and neuroimaging measures.

**Objectives:**

Here, we depict preliminary results regarding the epidemiological sample.

**Methods:**

We assessed internalizing and externalizing symptoms in 40 italian subjects (25 F) from general population at three waves (W1 mean age: 12±0,82; W2 mean age: 17±0,88, W3 mean age: 28±1), through the Child Behavior Checklist (W1 and W2) or the Adult Self Report (W3), and perinatal risk factors through a socio-anamnestic questionnaire, by a new online platform (MedicalBit). We analyzed symptoms trajectories and their relation with perinatal risk factors through a repeated measures multivariate analysis of variance (rm-MANOVA).

**Results:**

rm-MANOVA results show that high number of perinatal risks was significantly associated with higher internalizing symptomatology in preadolescence but not in adolescence and adult life. The mean difference was 8 T-points. The same trend is evident in adolescence but not in adult age (Graph 1). Perinatal risk factors did not have a significant effect on externalizing symptoms at any time point, despite a non-significant trend is evident (Graph 2).

**Figure:**
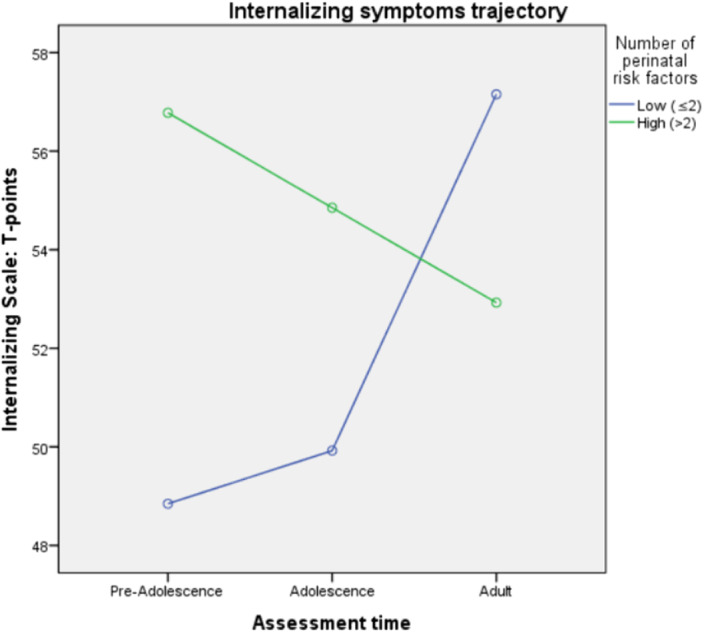

**Figure:**
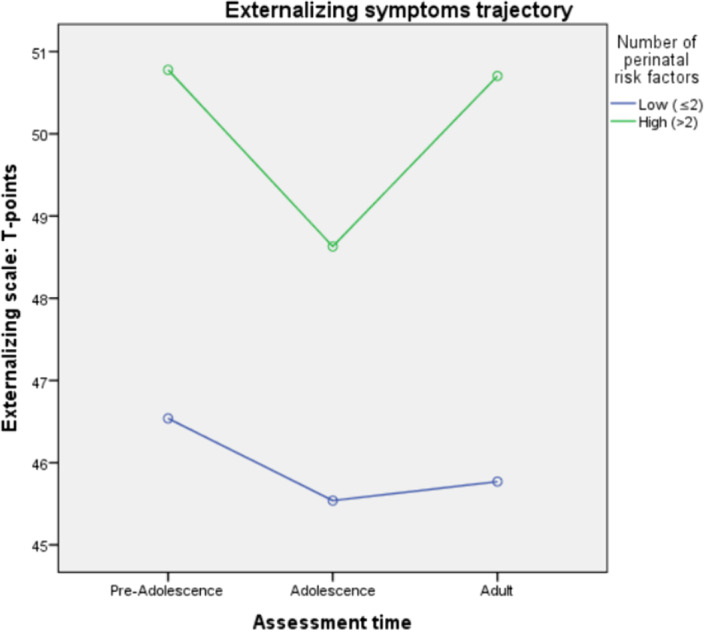

**Conclusions:**

Our preliminary results suggest a trend of increased internalizing symptoms from childhood to adulthood and a significant role of perinatal risk factors in pre-adolescence. Further investigations are necessary to better understand symptoms trajectories and the role of biological and environmental factors.

**Disclosure:**

No significant relationships.

